# Biofilm Formation, Antibiotic Resistance, and Infection (BARI): The Triangle of Death

**DOI:** 10.3390/jcm13195779

**Published:** 2024-09-27

**Authors:** Vincenzo Giordano, Peter V. Giannoudis

**Affiliations:** 1 Serviço de Ortopedia e Traumatologia Prof. Nova Monteiro, Hospital Municipal Miguel Couto, Rua Mário Ribeiro 117/2º Andar, Gávea, Rio de Janeiro 22430-160, RJ, Brazil; 2 Academic Department of Trauma and Orthopaedics, School of Medicine, University of Leeds, Leeds LS2 9LU, UK; 3 NIHR Leeds Biomedical Research Center, Chapel Allerton Hospital, Leeds LS7 4SA, UK

**Keywords:** biofilm formation, antibiotic resistance, infection, fracture-related infection, mortality, mortality rates

## Abstract

Fracture-related infection (FRI) is a devastating event, directly affecting fracture healing, impairing patient function, prolonging treatment, and increasing healthcare costs. Time plays a decisive role in prognosis, as biofilm maturation leads to the development of antibiotic resistance, potentially contributing to infection chronicity and increasing morbidity and mortality. Research exploring the association between biofilm maturation and antibiotic resistance in orthopaedics primarily addresses aspects related to quality of life and physical function; however, little exists on life-threatening conditions and mortality. Understanding the intrinsic relationship between biofilm maturation, bacterial resistance, and mortality is critical in all fields of medicine. In the herein narrative review, we summarize recent evidence regarding biofilm formation, antibiotic resistance, and infection chronicity (BARI), the three basic components of the “triangle of death” of FRI, and its implications. Preoperative, perioperative, and postoperative prevention strategies to avoid the “triangle of death” of FRI are presented and discussed. Additionally, the importance of the orthopaedic trauma surgeon in understanding new tools to combat infections related to orthopaedic devices is highlighted.

## 1. Introduction

Surgical site infection after fracture fixation, or more contemporaneously, fracture-related infection (FRI), which encompasses all infections occurring in the presence of a fracture, is a devastating event, directly affecting fracture healing, impairing patient function, prolonging treatment, and increasing healthcare costs [1,2,3]. The estimated incidence of FRI ranges from 1% to 2% in closed fractures, increasing up to 30% in Gustilo et al. grade IIIB open fractures [1,4]. The diagnosis is sometimes not simple, mainly due to the absence of the external signs of infection, but also due to the inability of the surgeon who operated on the patient to recognize signs of FRI. Confirmatory and suggestive diagnostic criteria were proposed by the FRI Consensus Group in 2018 and were recently validated, confirming the excellent diagnostic discriminatory value of them [3,5,6,7].

The consequences of FRI can be catastrophic, including long-lasting mental disease and depression, limb dysfunction, amputation, and even death. In fact, despite all improvements in diagnostic approaches, antibiotic drugs, and surgical techniques, especially in the last 10 years, the amputation and recurrence rates of infection still remain unacceptably high [6].

Although FRI is typically caused by direct inoculation from trauma, either due to the trauma itself, during implant insertion, or due to impaired wound healing or poor soft tissue coverage, its incidence and risk factors differ among fracture sites, hosts, environments, and pathogen types [2,4,7]. Polymicrobial infections occur in approximately 20% to 35% of the cases, mainly after open fractures [7].

The pathophysiology of FRI is multifactorial, with a vicious cycle that begins with bacterial infection and continues with biofilm formation, fracture instability, canalicular invasion, intracellular infection, antibiotic resistance, and chronic infection [1]. In this scenario, time plays a decisive role in prognosis, and as a rule, infections that have been in place for a longer period of time are more difficult to treat due to the maturation of biofilm, decreased or lost effectiveness of antimicrobial treatment, extensive bone necrosis, and life-threatening risk, particularly in the elderly [6,7,8,9,10].

In the herein narrative review, we summarize recent evidence regarding biofilm formation, antibiotic resistance, and infection chronicity (BARI), which are the three basic components of which we call the “triangle of death” of FRI, and their implications, including mortality.

## 2. Triangle of Death of Fracture-Related Infection

### 2.1. Biofilm Formation

A biofilm is a safe and antibiotic-resistant home to microorganisms, such as bacteria, that are capable of living and reproducing collectively within an extracellular polymeric substance matrix, forming colonies [11,12]. This living biomass has a sophisticated social structure that serves both to protect and allow the expansion of the colony, helping the maturation and dispersion of the microorganisms to start a new cycle of biofilm formation [11,12]. Ultimately, in a clinical setting, biofilm formation protects the colony of bacteria from antibiotic drugs, creating an environment of antibiotic resistance and greatly complicating the treatment of FRI. Staphylococcus aureus is the leading pathogen in FRI, responsible for 80% of human chronic osteomyelitis, with over 50% of the cases caused by methicillin-resistant *S. aureus* strains [13,14]. Other bacteria frequently found in a biofilm are *P. aeruginosa*, *S. epidermidis*, *E. coli*, *K. pneumoniae*, *P. mirabilis*, *S. viridans*, and *E. faecalis* [11,15].

At the molecular level, almost all bacteria use microbial surface components recognizing adhesive matrix molecules (MSCRAMMs), surface proteins that adhere to collagen and fibronectin on bone and allow for biofilm formation [4]. The mechanism of biofilm formation is a multistep and complex process that involves the transition of bacteria from a free planktonic form to a biofilm-making sessile form [11]. In fact, the adherence of planktonic microorganisms to surfaces is considered an important stage to develop the free-flowing microorganisms into an assembled community structure [11,16]. During the initial stage, Bis-(3′-5′)-cyclic dimeric guanosine monophosphate (c-di-GMP), an intracellular signalling molecule, plays an important role in the lifestyle changes of many bacteria by restricting flagella-mediated swimming motility, increasing biofilm matrix production, and regulating the biofilm transition from the motile to the sessile state [11,17]. Also, c-di-GMP controls the ability of bacteria from interacting with abiotic surfaces or with other bacterial and eukaryotic cells [17].

Sessile bacterial biofilm communities present different rates of growth, gene expression, transcription, and translation, thus facilitating their adaptation to microenvironments that have higher osmolarity, scarcer nutrients, and greater cell density [12]. After successful bacterial adhesion and aggregation, a new stage begins with the multiplication and cell division of bacteria with the formation of micro- and macro-microcolonies. In this context, the exopolysaccharide layer plays a crucial role in biofilm maturation, in particular, in protecting the community from the host’s immune response and antimicrobial drugs, and encapsulating signalling molecules necessary for quorum sensing, metabolic products, and enzymes [18]. The quorum sensing-dependent process allows bacteria to recognize the dimensions and proximity of adjoining groups, thus aiding in the formation of clusters that bond with nearby cells more effectively [11,12,19].

After colony maturation, the biofilm presents an internal regulatory layer, an intermediate microbial basal layer, and an external layer inhabited by the planktonic form of microorganisms, which are ready to leave the biofilm, beginning the dispersion phase [11,20,21]. The oxygen concentration near the surface of the biofilm is the highest, decreasing towards the centre, almost creating an anaerobic condition [22]. In this phase, individual or aggregated bacterial cells from the external layer spread to other areas of the implant and bone, including invading the osteocyte lacuno-canalicular and bone cells, and to other regions of the host to obtain nutrients and expel stress-inducing situations and waste by-products, initiating a new phase of biofilm formation [4,11,12,13,14,21,23,24]. In the remaining bacterial community, low oxygen conditions and intracellular c-di-GMP signalling reduce the metabolic state and virulence, which increases its stability and resilience, resulting in less penetration and consumption of antibiotics, leading to the development of antibiotic resistance and contributing to the chronicity of the infection [17,25].

### 2.2. Antibiotic Resistance

Although antibiotics are generally effective on susceptible bacteria and in the planktonic mode, antibiotic-resistant bacteria are not killed planktonically or outside the biofilm [25,26]. Resistance to antibiotics in biofilm communities and the persistence of biofilm infections despite antibiotic exposure occur due to multiple innate (phenotypic resistance) and acquired (genetic resistance) tolerance mechanisms [26].

The bacteria’s innate properties and wild-type genes associated with the restricted penetration of antibacterials through the biofilm matrix are the first mechanisms of defence. Overall, the innate resistance of the bacteria and the environmental structure of biofilms reduce antimicrobial diffusion and adsorption on the self-produced protective matrix of the exopolysaccharide layer and potentially increase clonal interference, rendering selection less effective and enhancing genetic diversity [26]. This tolerance is multifactorial, being mainly attributed to the restricted penetration of antibiotics through the biofilm, restricted growth at a low oxygen tension, an altered chemical microenvironment, the expression of biofilm-specific genes, and the presence of a subpopulation of persister micro-organisms, which are a dormancy state of a bacterial subpopulation, prevalent in the stationary state of biofilm communities, which is a slow or non-growth phase of the bacterial life cycle [25,26]. The formation of persister cells has been associated with the actions of toxins such as MazF and RelE from toxin–antitoxin (TA) modules and has been shown to be responsible for the recalcitrance of chronic infections, as they remain viable and are capable of repopulating biofilms when the level of antibiotics decreases [27,28]. Noteworthy, both MazF and RelE are toxin–antitoxin systems found on the chromosomes of Escherichia coli and other bacteria. MazF is a type II toxin–antitoxin system and RelE is a toxin that severely inhibits cell growth and colony formation.

The metabolic state and activation of resistance genes are consequences of the multicellular nature of biofilms and the excessive use of antibiotics and are indispensable for the development of antibiotic resistance in biofilm communities [25,26,27,28,29]. In fact, it has been demonstrated that acquired resistance by the bacteria results both from the exposure of planktonic forms to subinhibitory or progressively increasing the concentrations of antibiotics and from the use of antibiotic treatments for short periods [29]. The rate and extent of the evolved resistance depends on the strength of the antibiotic selection, the distribution of the fitness effects of mutations that increase drug resistance, and the size of the population of the replicating bacteria [25,26,27,28,29]. The stronger the selection for resistance, the greater the likelihood of genetic parallelism between replicate populations [29]. This mechanism of resistance is upregulated when biofilms are exposed to antibiotics and downregulated when the antibiotic molecules disappear from the infection site due to the metabolization and elimination of the antimicrobial molecules [30]. The occurrence of several heritable resistance mutations increases bacterial survivability and favours the emergence and selection of antibiotic-resistant mutants, both at the site of biofilm infection and systemically, playing a role in biofilm recalcitrance to antibiotic treatment, the chronicity of infection, and the risk of systemic complications, including sepsis, multiple organ failure, and death [25,26,27,28,29].

### 2.3. Infection and Its Impact

**Figure 1:**
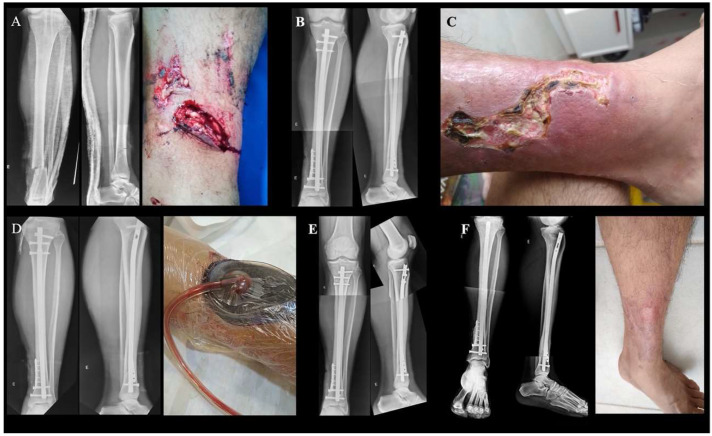
(**A**) 53-year-old man was involved in a motorcycle accident and sustained an open fracture on the left tibia. The patient reported having insulin-dependent diabetes. (**A**) AP and lateral radiographs showed a simple fracture of the distal tibia, but the degree of soft tissue involvement and severe contamination led the injury to be classified as Gustilo et al. grade 3A. (**B**) The patient was taken to the operating room (OR) for urgent irrigation and debridement, and immediate definitive fixation with a medial mini-fragment reduction plate and a reamed tibial intramedullary nail (IM). (**C**) Ten days after hospital discharge, the patient returned to the outpatient clinic complaining of pain in the operated leg and showing clear signs of acute FRI. (**D**) The patient was re-admitted to the hospital and taken to the operating room for wound irrigation and debridement. Samples were collected for culture. Due to the acute nature of the FRI, it was decided to initiate appropriate suppressive antibiotic therapy and maintain the IM implant. A negative pressure dressing was placed. (**E**) AP and lateral radiographs of the left leg taken 90 days after the second procedure showed uneventful healing of the fracture. (**F**) AP and lateral radiographs of the left leg after 2 years of follow-up showed no signs of infection and adequate healing of the soft tissues.

As previously stated, FRI is multifactorial, which makes its treatment challenging [1]. Time is the cornerstone in the pathogenesis of FRI as biofilm maturation plays a definitive role to differentiate between acute and chronic infections [7,31]. Generally, this cut-off point is six weeks to acute infections in the presence of internal fixation devices, with authors finding that up to two-thirds of the patients who managed with debridement, appropriate suppressive antibiotic therapy, and implant retention (DAIR) achieve uneventful fracture healing [32,33,34]. Figure 1 shows the case of a patient managed with DAIR. In general, after this period, DAIR is neither possible nor recommended in most cases due to a higher infection recurrence rate.

The chronicity of FRI due to biofilm formation and antibiotic resistance is a complication that impacts the costs of care, quality of life, and patient function and should be viewed as an important public health problem worldwide [6,7,30,31,35,36]. It has been shown that direct costs lead to a 2.5- to 8-fold increase in the total cost of medical care compared to uninfected patients, mainly related to the increased procedural costs and length of hospital stay [36,37,38,39,40]. In addition, prolonged absenteeism has been shown to indirectly contribute to the economic impact in patients with FRI [34]. In a non-concurrent cohort based on the retrospectively collected data of patients undergoing intramedullary nailing for tibial shaft fractures, Galvain et al. [37] noted that the average total costs were estimated to be GBP 14,756 for patients with infection versus GBP 8279 for those without infection. Iliaens et al. [38] in a matched-pairs analysis of patients with and without FRI found that the average hospital-related direct costs were EUR 47,845 for patients with FRI, compared with EUR 5983 for patients without FRI. Finally, in a retrospective case-control analysis comparing 21 patients with FRI and 63 uninfected patients, Woffenden et al. [40] noted that the total cost of healthcare was GBP 22,058 for patients with FRI, compared with GBP 8798 for the uninfected group.

The quality of life and physical function are also significantly affected. Walter et al. [39] in a retrospective cohort study with 37 patients with long bone FRI concluded that the quality of life was significantly reduced, especially in the physical health component, with a moderate to severe psychological symptom burden in up to 20% of the cases, even after an average of 4.2 years after successful treatment. In this scenario, chronic osteomyelitis undoubtedly represents the main cause of the reduced quality of life and physical impairment in patients with persistent FRI, as the incidence of relapse following an apparently ‘successful’ treatment remains high in these patients [41]. Hotchen et al. [42] assessed bone involvement, antimicrobial options, soft tissue coverage, and host status (BACH) classification as a prognostic tool and its ability to stratify cases of long bone osteomyelitis. They observed that the mean self-reported three-level EuroQol five-dimension questionnaire index score and visual analogue scale were significantly lower in patients with complex long-bone osteomyelitis compared to what they called uncomplicated osteomyelitis cases. In fact, in a systematic review of 93 studies describing 3701 patients (3711 fractures) with complex FRI, Bezstarosti et al. [43] observed a recurrence rate of infection in 9%, non-union in 7%, and amputation in 3% of all cases, which ends up impacting the quality of care for patients suffering from this devastating problem. Factors associated with persistent non-union, amputation, or other complications after open reduction internal fixation include time from injury to index surgery, poor nutritional status, obesity, smoking, open fractures, polymicrobial infections, culture-negative infections, and intramedullary implants [6,7,44,45]. Of interest, sustaining an amputation or open fracture, having an inpatient infection, and the use of anti-pseudomonal penicillin for more than 6 days were independently associated with the risk of an extremity wound infection among military personnel [46].

From the patients’ perspective, the chronicity of FRI has severe restrictions in their day-to-day life, with negative impacts on their emotional and mental status, expressed by anxiety and fears even after a successful surgery [47]. In addition, patients refer several socioeconomic consequences, mainly related to unemployment, divorce, and family disruption [47].

Avoiding the formation of biofilm and antibiotic resistance are fundamental measures to reduce the poor outcomes observed with the chronicity of the infection; the orthopaedic trauma surgeon plays a leading role in preventing and treating the triad of death of FRI [36,45].

## 3. Materials and Methods

A literature review using a computerized search of the PubMed Medline database was carried out. The common keywords ‘antibiotic resistance’ and ‘mortality’ were used to identify papers which discussed or investigated any relationship between BARI and mortality. The search included a title and abstract containing the following Boolean terms: ((fracture-related infection) OR (mortality rates) AND (biofilm formation) OR (antibiotic resistance) AND (infection)). This was complemented by a manual search of relevant citations from articles retrieved via Google Scholar in order to identify the most current evidence on the relationship between antibiotic resistance and mortality. The bibliography of each retrieved article was assessed for additional relevant studies. Citations from relevant systematic reviews and meta-analyses were searched for additional studies of interest.

Articles were selected that met the following inclusion criteria: (1) contain data on the influence of biofilm formation and/or antibiotic resistance and/or chronicity of infection on the mortality rate; (2) clinical study, comparative study, controlled clinical trial, evaluation study, meta-analysis, multicentre study, observational study, pragmatic clinical trial, randomized clinical trial, and systematic review; (3) published in the last 5 years; (4) carried out in humans; (5) published in English; and (6) carried out on adults over 19 years old. The exclusion criteria were: (1) studies containing insufficient data regarding the association between biofilm formation, antibiotic resistance, and mortality rate; and (2) a lack of information on mortality rate either as a primary or secondary outcome in data collection and analysis or in the presentation of the main results; (3) studies reporting fungal infections, COVID-19, and/or cancer diseases, (4) studies on new therapeutic approaches or comparative antibiotic treatments, and (5) median follow-up of less than 30 days in clinical trials, clinical research, and systematic reviews. Relevant information about the year of publication, journal name, author name, the type of study, the influence of biofilm formation and/or antibiotic resistance and/or infection on mortality rate was carefully extracted.

Based on the titles and abstracts, the investigators (V.G. and P.V.G.) picked out the potential eligible studies. All duplicate titles were removed. Then, the full text of the remaining studies was reviewed for eligibility by the same investigators, and any disagreements were resolved by discussions between authors for the final decision. All studies were independently assessed to check whether they met the inclusion criteria. Again, if there was a disagreement regarding inclusion, it was resolved by a discussion involving both authors for the final decision, and if no consensus could be reached, then the study was excluded.

Each primary study was assessed based on its OCEBM Level of Evidence (LoE) [48]. The potential presence of publication bias was firstly explored visually by generating the respective funnel plots for the main outcomes of interest. A symmetrical distribution of the studies about the pooled effect estimate would be interpreted as an absence of publication bias. Furthermore, we utilised the Egger’s test and the Begg’s rank test. For both tests, there is an indication of publication bias when the two-sided *p*-value is very low (below the significance level). Sensitivity analysis was performed by repeating the pooling process after eliminating studies of either low methodological rating by the MINORS tool, or dubious eligibility [49]. Should this process not yield considerably different results than the originally obtained, our confidence surrounding the robustness of our findings would increase. The risk of bias was assessed using the Cochrane robvis visualization tool and evaluated according to the Risk of Bias in Non-randomized Studies—of Interventions (ROBINS-I) tool for studies not randomized [50].

## 4. Results

**Figure 2:**
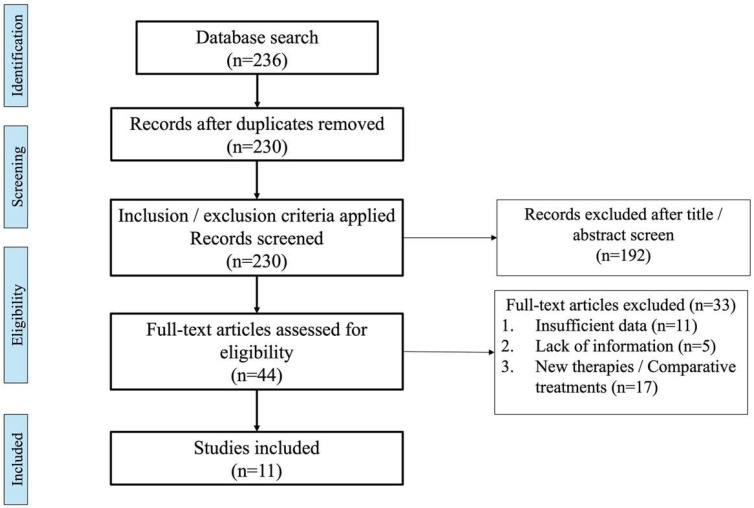
PRISMA (preferred reporting items for systematic reviews and meta-analyses) flowchart displaying the number of studies retrieved following searches and removed at each screening stage.

A total of 236 records were identified through a database search. After screening titles and abstracts and removing duplicates, 44 potentially relevant records met our eligibility criteria, and full-text articles were selected. Of these, 33 records were discarded, leaving 11 records that were included in the final review [51,52,53,54,55,56,57,58,59,60,61]. The search strategy for databases with a flowchart of the literature selection process is outlined in Figure 2.

Six (54.5%) studies were observational retrospective reviews (grade of recommendation A and level of evidence Ib), two (18.2%) studies were systematic reviews (grade of recommendation B and level of evidence III), two (18.2%) studies were a retrospective cohort (grade of recommendation B and level of evidence III), and one (9.1%) study was a retrospective, multicentre analysis (grade of recommendation B and level of evidence IIb). These are summarised in Table 1.

All studies were assessed using the MINORS criteria, which contains twelve items, the first eight being specifically for non-comparative studies, giving a maximum score of 16 for non-comparative studies and 24 for comparative studies. A maximum score indicates that methodological items were adequately reported. The mean score was 11.4 (ranged from 11 to 13) for non-comparative studies and 17.5 (ranged from 15 to 20) for comparative studies. The methodological index for non-randomized studies (MINORS) criteria is presented in Table 2.

**Figure 3:**
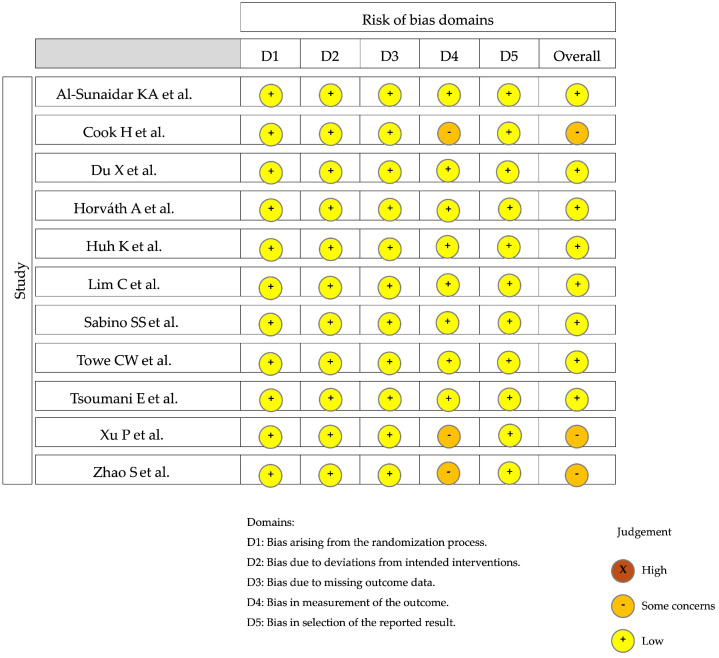
Results of the risk of bias in non-randomized studies—of interventions (ROBINS-I) tool for studies not randomized, which are visualized in traffic light plots for each individual domain assessed, using Cochrane robvis visualization tool [51,52,53,54,55,56,57,58,59,60].

An unbiased assessment of the study endpoint and the lack of a prospective calculation of the study size were the main problems encountered in the non-comparative studies, whereas an unbiased assessment of the study endpoint, the lack of a prospective calculation of the study size, and inadequate statistical analyses were the main problems of comparative studies. The risk of bias is shown in Figure 3 [50].

## 5. Discussion

Understanding the intrinsic relationship between bacterial resistance and mortality is critical in all fields of medicine. According to the last report by the Director-General of the World Health Organization (WHO), an estimated 1.27 million global deaths were attributed to drug-resistant bacterial infections in 2019, with low- and middle-income countries most affected [62,63]. Of note, in Latin America, multidrug-resistant microorganisms are the leading cause of healthcare-associated infections, with significant consequences for health systems in terms of mortality, disability, and economic costs [64]. This is highly driven by the misuse and overuse of antimicrobials, and inappropriate empirical antimicrobial treatment [52,63]. Indeed, in a systematic review of 19 observational studies, Du et al. [53] observed that inappropriate empirical antimicrobial treatment significantly increased the mortality rate of patients infected with carbapenem-resistant *A. baumannii* (CRAB). In another study, Al-Sunaidar et al. [51] retrospectively analysed 228 adult patients admitted to the ICU ward with a diagnosis of sepsis or who manifested sepsis symptoms and the mortality rate in patients who received an appropriate empirical antibiotic was 39% lower than that in patients with non-appropriate empirical antibiotics.

A common mechanism of antibiotic resistance in bacteria involves the production of an enzyme that modifies or destroys the structure of antibiotics by inactivating them, either through structural changes in the enzymes involved in cell wall biosynthesis and the synthesis of nucleic acids and metabolites, or an enzymatic modification of the structural elements affected by antibiotics [62]. Mutations in bacterial genomes and the selection of new resistant phenotypes significantly increase substrate specificity and provide an evolutionary advantage for extremely broad resistance to various antibiotics [52,65]. Mutations can affect how these resistances are expressed, and once a mutation that can potentially generate an antibiotic resistance phenotype has occurred, the bacteria carrying the mutated allele must compete with the wild-type ancestor bacterial population [66]. The probability of an effective resistance mutation depends on the structure and the number of the genes in which mutations can produce a selectable phenotype [65,66,67]. Some authors have demonstrated in isolates from different species that a variety of genes are involved in antibiotic resistance, mainly due to the different pathways of targeting, access, and protection for the antibiotic in the bacterial cell [63,64]. A combination of several resistance mechanisms in a single cell and transferring the genes coding for these enzymes to other bacteria complicate the development of methods for suppressing resistance and increase the mortality rate [62]. Horváth et al. [54] observed that 30-day all-cause mortality was significantly increased in patients with methicillin-resistant *S. aureus* (MRSA) bloodstream infections caused by the Staphylococcal cassette chromosome mec (SCCmec) type IV, which plays a significant role in the evolution and diversification of acquired resistance determinants to this specific pathogen.

Other risk factors for antibiotic resistance and increased mortality have been identified, such as older age, pre-existing comorbidities, some specific underlying conditions, prolonged hospitalization, current or previous antibiotic use, a longer duration of antibiotic therapy, and existing sites of infection, with the mortality rate reaching up to 50% of the cases in some studies [51,52,53,55,56,57,59,61,68]. In a systematic review by Tsoumani et al. [59] including 82 studies published from 2011 to 2021 that sought to summarize the clinical, economic, and humanistic burden of CAP in Europe, the mortality at 30 days ranged from 39 to 44.5%, and antibiotic resistance was directly related to this finding.

Several authors have been reporting the close relationship between antibiotic resistance and increased mortality rates. In a retrospective cohort study using data from a South Korean database to determine the impact of difficult-to-treat on the 30-day in-hospital mortality of patients with a Gram-negative bloodstream infection, Huh et al. [55] found a mortality rate of 50.3%. In another study comparing antimicrobial-resistant bloodstream infections in tertiary-care hospitals compared with secondary-care hospitals in Thailand, Lim et al. [56] observed a mortality rate following community-origin and hospital-origin antimicrobial-resistant bloodstream infections of 27.5% (2873 out of 10,858 patients) and 38.2% (3874 out of 8807 patients), respectively. Sabino et al. [57] observed a mortality rate of 37.8% among adult patients who developed healthcare-associated infection and were admitted to intensive care units of a university hospital in Brazil, mostly due to resistant/multidrug-resistant pathogens. In another study that analysed the association of microbiology and antibiotic resistance with postoperative mortality after the decortication for empyema, Towe et al. [58] reported a 90-day mortality of 7.6% (14 of 185 patients), with a trend toward higher resistance counts and adverse outcomes.

Another mechanism of antibiotic resistance is the failure to control biofilm-related diseases, which increase infection-related morbidity and mortality rates [65,69]. Several studies have shown that biofilm maturation and the multidrug resistance of biofilms in medical devices and implants make the distribution of extracellular products highly non-uniform, thus the use of antibiotics alone to treat infections brought on by biofilm is ineffective [65,69,70,71,72]. In some bacteria, such as *P. aeruginosa* and *S. aureus*, the outer slime layer provides stability to the biofilm and helps transport nutrients through its pores, while the inner layer increases antibiotic resistance by supporting the transfer of genetic information [12,69,71].

Therefore, especially in acute infections caused by multidrug-resistant species or chronic infections, internal medical devices or living tissues can be rapidly contaminated, making it difficult to eradicate the biofilm due to a high tolerance to antibiotics and increasing the mortality rate [73,74]. In this scenario, the most effective treatment for biofilm-related infections is to debride and resect the infected tissue in combination with antibiotic therapy, in addition to removing the infected medical device when necessary [75]. For example, postoperative chronic osteomyelitis has been shown to represent a major health problem due to its increased long-term mortality risk, especially in certain populations and skeletal sites [76,77,78]. Huang et al. [77] found that chronic osteomyelitis had a significantly higher mortality risk than those without chronic osteomyelitis, particularly the elderly (≥85 years) and males. The same was observed by Yagdiran et al. [78], who found older patients, male patients, and patients with comorbidities to be at a higher risk of death due to vertebral osteomyelitis. These authors observed mortality rates of 20% and 23% at 1 and 2 years, respectively, in 195 patients treated surgically for vertebral osteomyelitis. Dudareva et al. [79] observed 12 deaths in 61 adults with chronic pelvic osteomyelitis treated with combined medical and surgical treatment. They advocate that a multidisciplinary approach allows successful treatment in most cases. A similar multidisciplinary approach was proposed by Cook et al. [52] in 51 patients presenting heel ulcerations and calcaneal osteomyelitis treated with a vertical contour calcanectomy. They reported a total limb salvage rate of 68.6%. All-cause mortality was reduced to 9.8% at one year.

The economic burden of the triangle of death is enormous on healthcare environments due to the high morbidity and mortality rate, especially associated with multidrug-resistant bacteria. In a systematic review of 20 studies, Serra-Burriel et al. [80] found that multidrug-resistant healthcare-associated infections were strongly associated with excess attributable costs, prolonged length of stay, and increased mortality. In another systematic review of 29 studies, 20 of these conducted in high-income countries and the remainder in upper- and middle-income countries, Poudel et al. [81] observed that the attributable costs for resistance infections compared to susceptible infections ranged from (negative) − USD 2371.4 to USD 29,289.1. The mean excess mortality was 6.9% (20 studies), with patients presenting a resistant infection having an 84.4% more chance of dying compared to patients with a susceptible infection. Hence, the triangle of death must be considered and managed as a major public health concern, since it contributes significantly to increased healthcare costs for both patients and providers, which makes preventive strategies imperative.

### Prevention Strategies

FRI prevention measures can be divided into preoperative, perioperative, and postoperative stages [31,36]. During all these stages, the orthopaedic trauma surgeon must have the collaboration of specialized nurses, infectious disease specialists, clinicians, and anaesthetists, as the work of a multidisciplinary team has been shown to improve patient safety, quality of care, and results, which reduces healthcare costs [40,45,82]. Preventing biofilm formation and maturation and antibiotic resistance is the cornerstone to reducing the risk of conical infection and the installation of the FRI death triangle.

Preoperative measures include assessing the general and local health status of patients, correcting potential factors that compromise the patient’s general health status and increasing the risk of postoperative complications [36,45,82]. In addition, screening tests must be carried out to detect community microorganisms, such as Staphylococcus aureus, present in up to 17% of trauma patients [36,82]. In fact, some comorbidities cannot be optimized at this stage, such as obesity, malnutrition, or smoking, but must be addressed in the later stages of treatment.

Perioperative measures include the careful preparation of the surgical site, administration of prophylactic antibiotics in a single dose 30 to 60 min before incision, adequate surgical environment, and refined and less traumatic surgical technique with careful manipulation of all tissues, especially the soft tissue envelope. Current therapeutic interventions to combat implant-related bone infections, either using local antibiotic carriers or nanocomposites capable of regulating cellular function, have been evaluated in animal models with promising results [83,84,85]. A recent systematic review of 43 studies, of which 10 specifically dealt with fracture-related infections, showed that combining local antibiotic therapy and the systemic administration of antibiotic agents effectively reduced biofilm at early stages [84]. Comparing the antibiotic hydrogel with systemic perioperative isolated antibiotic prophylaxis in an animal model using sheep inoculated with methicillin-sensitive *Staphylococcus aureus* at the time of the insertion of the intramedullary nail into the tibia, Boot et al. [83] demonstrated that locally administered antibiotic hydrogel is effective for preventing orthopaedic device-related infections compared to systemic antibiotics alone. Hydrogels have been shown to broaden the range of the activities of antimicrobial treatments and can deliver different antibiotics to the implant region. The main advantage of this procedure is the correct selection and dosage of the antibiotic to be applied to the biomaterials used [86]. Furthermore, hydrogel resorption was shown not to interfere with osteointegration, in addition to being sensitive and responsive to a variety of stimuli, including the presence of bacteria [86,87].

Topical intrawound vancomycin powder has been tested to determine whether it changes the bacteriology of surgical site infection pathogens. The VANCO Trial showed that topical vancomycin powder decreased the likelihood of Gram-positive infections consistent with the biologic activity of vancomycin in 29 patients who became infected after a fixation of tibial plateau or pilon fracture [88]. They observed fewer methicillin-susceptible *S. aureus* and coagulase-negative Staphylococci infections in the group treated with vancomycin powder (*n* = 29) compared to the control group (standard of care with no antibiotic powder, *n* = 45). There was no emergence of Gram-negative rod infections or increased resistance patterns observed. In another study from the same group, 980 adult patients with a tibial plateau or pilon fracture deemed to have a high risk of infection and definitively treated with plate and screw fixation, were randomly allocated in a 1:1 ratio to receive 1000 mg of intrawound vancomycin powder at their definitive fixation or to a control group that received no topical antibiotics [89]. Of these, 874 patients had at least 140 days of follow-up. Deep surgical site infections occurred in 30 patients (6.9%) in the vancomycin group and 48 patients (10.9%) in the control group. The estimated probability that intrawound vancomycin powder reduces the risk of a deep surgical site infection was >98%. Although these studies have shown interesting findings using topical intrawound antibiotic powders, it should always be kept in mind that the resistance of Gram-positive bacteria to glycopeptide antibiotics is caused by the production of enzymes that catalyse the modification of peptidoglycan in the bacterial cell wall [75]; therefore, further studies are needed, eventually using different antimicrobial drugs.

Another interesting therapeutic intervention is the use of antibiotic-coated implants. Antibacterial coatings on the surface of materials have become an important way to inhibit initial bacterial adhesion and subsequent biofilm formation through the steric exclusion of hydrophilic macromolecules on the surface of the orthopaedic implants [90]. Surface modification by polyethylene glycol (PEG) and zwitterionic polymers are the most studied strategies to prevent the adhesion of proteins and microorganisms on the surface of biomaterials, since they have demonstrated the ability to adsorb a large amount of water, forming a stable hydration layer [90,91]. The hydrophilicity of PEG, the dynamic movement of PEG chains attached to the surface, and the lack of binding sites make it difficult for bacteria and other microorganisms to adhere to the coating surface, while zwitterionic polymers have stronger surface hydration properties, with stronger antibacterial adhesion effects compared to ion-free PEG [90]. Some studies have demonstrated the excellent resistance of anti-adhesive polymer coatings due to their strong hydrophilic properties in vitro, with up to a 95% prevention of bacterial adhesion in short-term evaluation [92,93].

Although the translation of these findings to the clinical setting is still quite challenging, mainly due to the observation that hydrophilic surface coatings, in addition to preventing unwanted bacterial adhesion, can reduce or even prevent cell adhesion, which is essential for osteogenesis and reducing the risk of implant failure [90], several authors have shown the benefits of local prophylactic antibiotic therapy using antibiotic-coated intramedullary nails, especially in open fractures of the tibia [94,95]. In addition, by reducing the risk of infection, gentamicin-coated nails indirectly save costs by reducing operating room time and the length of hospital stay, as well as reducing patient morbidity and mortality [96,97]. Franz et al. [96] reported a 75% lower infection rate and up to 15% cost savings in 193 patients with grade III open fractures managed at four European trauma centres with a 1-year time horizon.

Zamorano et al. [98] retrospectively evaluated a cohort of 243 patients with open tibial fractures, 104 patients treated with a gentamicin-coated tibial nail (Expert Tibial Nail PROtect™, DePuy Synthes, J&J Company), and 139 treated with a non-gentamicin-coated nail (Expert™ Tibial Nail, DePuy Synthes, J&J Company). The use of a gentamicin-coated tibial nail was associated with a significantly lower incidence of fracture-related infection at a follow-up of 12 months. In addition, there was a shorter time to healing and lower rate of non-unions in patients treated with a gentamicin-coated nails, suggesting a protective factor against tibial non-union. No adverse effects attributed to locally administered gentamycin were observed. Karupiah et al. [99] evaluated whether a novel noble metal nail-coating technology could prevent bacterial adhesion and biofilm formation without interfering with bony union. They operated on 35 consecutive patients either with an isolated tibial (*n* = 26) or femoral (*n* = 9) open fracture using an anti-infective noble metal alloy-coated titanium intramedullary nail (OrthoSyn™, Vigilenz Medical Devices). All patients had definitive surgery within 24 h of admission. Bony union was achieved in 93.8% patients, with three patients developing infection, which resolved after antibiotic therapy. No safety issues were recorded. Kotsarinis et al. [100] investigated the safety and early clinical outcomes of a noble metal-coated titanium tibial nail (ZNN™ Bactiguard, Zimmer Biomet) in 31 patients with tibial shaft fractures at an increased risk of developing complications. Eight patients were heavy smokers or intravenous drug users at the time of the operation, three patients were diabetic, two patients had hypertension, two patients had asthma, two patients had psoriasis, and one patient had hypothyroidism. In addition, two patients sustained polytrauma, five patients had an open fracture, and 13 had concomitant soft-tissue involvement (Tscherne grade 1 or 2). At a mean follow-up period of 14.3 months, they observed favourable outcomes with the Bactiguard-coated intramedullary nailing device, with 27 presenting uneventful healing with no further intervention in a mean time of 3.3 months. No adverse events were seen related to any local or systemic allergic reactions to the metal alloy.

The use of metal coated internal implants with gold, silver, or palladium has been a current trend in orthopaedics, as this generates a galvanic effect on contact with moisture, potentially preventing bacterial adhesion and biofilm formation [99,100]. Results from in vitro and in vivo studies showed that by varying the noble metal ratio at implant surfaces, it is possible to modulate inflammation and fibrosis in soft tissue [99,100,101,102]. Using polydimethylsiloxane sheets with a thickness of 1 mm only or coated with the noble metals silver, gold, and/or palladium, Suska et al. [102] observed that coatings of silver only or silver with medium amounts of gold and low-medium palladium content were associated with a decreased recruitment of inflammatory cells to implant close exudates, a lower percentage of neutrophils, higher cell viability, and a lower production of monocyte chemoattractant protein-1, compared to the other coatings and the uncoated polydimethylsiloxane control.

Postoperative measures include adequate and careful wound care, explaining to the patient how to do it at home, avoiding prolonged antibiotic therapy (prophylactic regimen only), preparing the patient for rapid hospital discharge, and quickly initiating a rehabilitation protocol [36]. The standard practice for prophylactic infection prevention is 24 h on clean wounds, while for open, contaminated wounds, it should not exceed 72 h [36,103].

## 6. Conclusions

**Figure 4:**
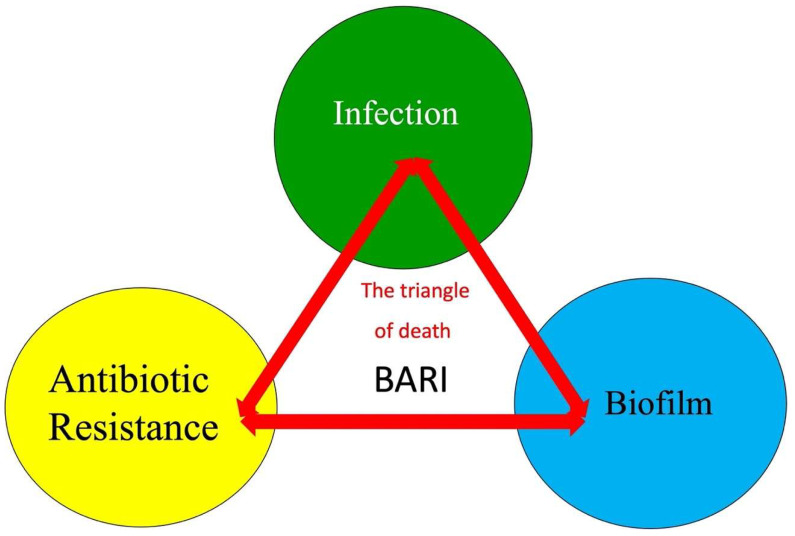
The triangle of death: biofilm formation (and maturation), antibiotic resistance, and infection (BARI).

Biofilm formation (and maturation), antibiotic resistance, and infection (BARI) form the triangle of death of FRI, with recognized disastrous consequences ranging from non-union and multiple surgical interventions to amputation (Figure 4).

Our study highlights the importance of preventing this triangle of death by preventing the spread of multidrug-resistant bacteria from the bone to the bloodstream, which potentially increases the risk of sepsis, multiple organ failure, and death. In addition to the physical repercussions, several emotional, social, and financial changes are perceived by patients, such as anxiety and depression, family disruption, and unemployment. No less important, the costs associated with the triangle of death of FRI are high and burden healthcare systems around the world. The role of the orthopaedic trauma surgeon is to reduce this burden through preventive strategies at all stages of treatment and understanding new tools to combat orthopaedic device-related infections.

## Figures and Tables

**Table 1:** Summary of included articles from the literature search.

Article Title	Publication Year	First Author	Journal	Article Type	Relevant Findings
Appropriateness of empirical antibiotics: risk factors of adult patients with sepsis in the ICU [51]	2020	Al-Sunaidar KA	Int J Clin Pharm	Observational retrospective	A total of 228 patients admitted to the adult ICU ward from 2011 to 2015 with a diagnosis of sepsis or who manifested sepsis symptoms were included. The most isolated microorganisms were Gram-negative bacteria (78%), and Gram-positive species comprised 22%. The total mortality rate was 193 (84.6%), with 119 males (52.2%) and 74 females (32.5%). The frequency of appropriate empirical antibiotics was 64 (28.1%). The mortality rate amongst patients who received appropriate antibiotics was 47 (20.6%), whereas amongst patients who did not was 146 (64%) (*p* = 0.007). Death in patients who received appropriate empirical antibiotic was 39% lower than that in patients with non-appropriate empirical antibiotics (HR 0.610, 95% CI 0.433–0.858, *p* = 0.005).
Early clinical, functional, and mortality outcomes for heel ulcers treated with a vertical contour calcanectomy [52]	2022	Cook H	J Foot Ankle Surg	Observational retrospective	A total of 51 patients suffering from chronic conditions and presenting heel ulcerations and calcaneal osteomyelitis were treated with a vertical contour calcanectomy. Here, 31.4% of patients had no recurrence, amputation, or mortality at 1-year follow-up. The total limb salvage rate was 68.6% and all-cause mortality was 9.8% at one year.
Predictors of mortality in patients infected with carbapenem-resistant Acinetobacter baumannii: a systematic review and meta-analysis [53]	2019	Du X	Am J Infect Control	Systematic review	This systematic review included 19 observational studies. Inappropriate empirical antimicrobial treatment was one of the major factors associated with the mortality of patients infected with Carbapenem-resistant Acinetobacter baumannii (CRAB), as well as the severity of baseline condition. Inappropriate empirical therapy increased 5-fold of the pooled mortality of 1169 patients (12 studies) with CRAB infection (*p* < 0.001).
Characterisation of antibiotic resistance, virulence, clonality and mortality in MRSA and MSSA bloodstream infections at a tertiary-level hospital in Hungary: a 6-year retrospective study [54]	2020	Horváth A	Ann Clin Microbiol Antimicrob	Observational retrospective	Antibiotic susceptibility, prevalence of virulence factors, genotype, and all-cause 30-day mortality of patients with MRSA and MSSA strains were compared from BSI over a 6-year period. A total of 306 *S. aureus* BSI isolates (153 MRSA and 153 MSSA strains) were analysed. Resistance rates of the MRSA isolates were significantly higher towards ciprofloxacin, erythromycin, clindamycin, amikacin, tobramycin, and gentamicin compared to MSSA isolates, whereas resistance rates of MSSA isolates were the highest to erythromycin and doxycycline. Almost all isolates were sensitive to sulfamethoxazole-trimethoprim and rifampicin. Of these, 81.7% of MRSA isolates were multidrug-resistant, whereas only 3.6% of MSSA isolates were multidrug-resistant. All-cause 30-day mortality was 39.9% in the MRSA and 30.7% in the MSSA group (*p* < 0.0001). Infections caused by SCCmec type IV isolates were associated with the highest mortality rate (42.2%), despite the similar comorbidity rates of the different patient groups.
Impact of difficult-to-treat resistance in Gram-negative bacteremia on mortality: retrospective analysis of nationwide surveillance data [55]	2020	Huh K	Clin Infect Dis	Observational retrospective	A total of 1167 episodes of monomicrobial Gram-negative BSI caused by 4 major taxa (*E. coli*, *K. pneumoniae*, *P. aeruginosa*, and Acinetobacter species) were identified from a nationwide surveillance database. Of these, 147 (12.6%) of the isolates were DTR (79.6% of Acinetobacter species and 17.7% of *P. aeruginosa*). DTR infections were associated with previous antibiotic use, healthcare contact, ventilator use, and lower respiratory tract infection. A total of 243 patients (26.3%) died in the hospital within 30 days of the onset of Gram-negative BSI. Crude mortality was significantly higher in patients with DTR Gram-negative BSI (*p* < 0.001). Mortality for Gram-negative BSI caused by DTR was 50.3%, whereas mortality among the other resistance categories was similar.
Frequency and mortality rate following antimicrobial-resistant bloodstream infections in tertiary-care hospitals compared with secondary-care hospitals [56]	2024	Lim C	PLoS One	Retrospective, multicentre analysis	The data of 19,665 hospitalised patients with AMR BSI caused by CRAB, CRPA, 3GCREC, 3GCRKP, CREC, CRKP, and MRSA were analysed. Of these, 10,858 (55.2%) were classified as community-origin BSI and 8807 (44.8%) were classified as hospital-origin BSI. Of 10,858 patients with community-origin AMR BSI, 2873 (27.5%) died, whereas of 8807 patients with hospital-origin AMR BSI, 3874 (38.2%) died. All-cause in-hospital mortality following hospital-origin AMR BSI was not significantly different between tertiary-care hospitals and secondary-care hospitals. CRAB had the highest mortality rate per 100,000 patient-days at risk in both tertiary-care hospitals and secondary-care hospitals.
Infections and antimicrobial resistance in an adult intensive care unit in a Brazilian hospital and the influence of drug resistance on the thirty-day mortality among patients with bloodstream infections [57]	2020	Sabino SS	Rev Soc Bras Med Trop	Retrospective cohort study	A total of 2168 patients were admitted in an ICU at a 3-year period and a total of 1979 (55.1%) healthcare-associated infection episodes were observed in these patients. Most nosocomial infections were acquired in the ICU (81.2%). Blood stream (33.4%), lung (30.5%), and urinary tract (16.6%) infections were the most observed. 1722 (87%) episodes were monomicrobial and 257 (13%) were polymicrobial. The most prevalent BSI agents were CoNS (45.2%), *A. baumannii* (8.7%), and *P. aeruginosa* (7.9%). The mortality rate among patients who developed healthcare-associated infection was 37.8%.
Antibiotic resistance is associated with morbidity and mortality after decortication for empyema [58]	2021	Towe CW	Ann Thorac Surg	Observational retrospective	A total of 185 patients who received surgical decortication for empyema were analysed for the association of microbiology and antibiotic resistance with adverse postoperative outcomes. A total of 118 (63.8%) underwent decortication for primary empyema and 67 (36.2%) for secondary empyema. Of the 185 decortications, 103 organisms were cultured from 79 (42.7%) patients. Gram-positive organisms were most common (60/79, 75.6%), being more frequently seen in patients with primary than secondary empyema (40/45 (88.9%) vs. 20/34 (58.8%), *p* = 0.002), while polymicrobial infections occurred in 17 patients (21.5%) and were more common among patients with secondary empyema (11/34 (32.4%) vs. 6/45 (13.3%), *p* = 0.042). The most common bacterial organisms were Streptococcus species (29/79, 36.7%), *S. aureus* (19/79, 24.1%), and Pseudomonas species (6/79, 7.6%). Antibiotic resistance was seen in 73 patients with positive cultures and was more common in patients with secondary empyema (*p* = 0.001). Among the 73 patients who demonstrated antibiotic resistance, 39 (53.4%) were resistant to at least one antibiotic. Mortality at 90 days occurred in 14 (7.6%) patients.
Clinical, economic, and humanistic burden of community acquired pneumonia in Europe: a systematic literature review [59]	2023	Tsoumani E	Expert Rev Vaccines	Systematic review	This systematic review included 82 studies published from 2011 to 2021 that sought to summarize the clinical, economic, and humanistic burden of CAP in Europe. The most frequently implicated bacterial pathogen was *S. pneumoniae* (implicated in 43% of cases) followed by *H. influenzae* and *S. aureus* (16.1% and 9.6%, respectively). Mortality at 30 days ranged from 39 to 44.5%, with the highest mortality being reported among elderly patients who were admitted for inpatient treatment for CAP. Another factor associated with higher mortality at 30 days was antibiotic resistance. Longer lengths of stay (approximately 19–23 days) were associated with multidrug resistance and admittance through the ICU.
Clinical features and risk factors for mortality in patients with *Klebsiella pneumoniae* bloodstream infections [60]	2024	Xu P	J Infect Dev Ctries	Observational retrospective	The clinical features and risk factors for mortality were investigated in 145 patients (121 in the survival group and 24 in the non-survival group) with *K. pneumoniae* BSI infections. The main sources of *K. pneumoniae* BSI were liver infection (24.1%), urinary tract infection (17.9%), and biliary tract infection (17.2%). The *K. pneumoniae* strain had the highest rate of resistance to ticarcillin (53.8%) and ciprofloxacin (43.8%). Multidrug resistance was higher in the non-survival group than in the survival group (41.7% vs. 16.5%, *p* = 0.005).
Risk factors for antibiotic resistance and mortality in patients with bloodstream infection of *Escherichia coli* [61]	2022	Zhao S	Eur J Clin Microbiol Infect Dis	Retrospective cohort study	The clinical data of 388 patients were analysed to investigate the risk factors for BSI caused by ESBL-producing *E. coli*. The prevalence of ESBL-producing *E. coli* in BSI patients was 40.98% (159 of 388). *E. coli* isolates were commonly susceptible to carbapenem and β-lactam/β-lactamase inhibitor combinations. Only 0.52%, 0.93%, and 2.84% of isolates showed in vitro resistance to imipenem, ertapenem, and piperacillin/tazobactam, respectively. Regarding non-carbapenem and non–β-lactam antibiotics, the highest resistance was recorded for ampicillin/sulbactam (49.52%) and the lowest resistance was recorded for amikacin (1.55%). ESBL positivity, nosocomial infection, and cancer were independent risk factors of mortality.

**Table 2:** Methodological index for non-randomized studies (MINORS) criteria.

Study	Methodological Index for Non-Randomized Studies (MINORS) Criteria
Appropriateness of empirical antibiotics: risk factors of adult patients with sepsis in the ICU [51]	11 (non-comparative study)
Early clinical, functional, and mortality outcomes for heel ulcers treated with a vertical contour calcanectomy [52]	11 (non-comparative study)
Predictors of mortality in patients infected with carbapenem-resistant *Acinetobacter baumannii*: a systematic review and meta-analysis [53]	20 (comparative study)
Characterisation of antibiotic resistance, virulence, clonality and mortality in MRSA and MSSA bloodstream infections at a tertiary-level hospital in Hungary: a 6-year retrospective study [54]	12 (non-comparative study)
Impact of difficult-to-treat resistance in Gram-negative bacteremia on mortality: retrospective analysis of nationwide surveillance data [55]	11 (non-comparative study)
Frequency and mortality rate following antimicrobial-resistant bloodstream infections in tertiary-care hospitals compared with secondary-care hospitals [56]	16 (comparative study)
Infections and antimicrobial resistance in an adult intensive care unit in a Brazilian hospital and the influence of drug resistance on the thirty-day mortality among patients with bloodstream infections [57]	11 (non-comparative study)
Antibiotic resistance is associated with morbidity and mortality after decortication for empyema [58]	13 (non-comparative study)
Clinical, economic, and humanistic burden of community acquired pneumonia in Europe: a systematic literature review [59]	19 (comparative study)
Clinical features and risk factors for mortality in patients with *Klebsiella pneumoniae* bloodstream infections [60]	15 (comparative study)
Risk factors for antibiotic resistance and mortality in patients with bloodstream infection of *Escherichia coli* [61]	11 (non-comparative study)

## Data Availability

The original contributions presented in the study are included in the article material. Further inquiries can be directed to the corresponding author.
